# Numerical Evaluation of the Elastic Moduli of AlN and GaN Nanosheets

**DOI:** 10.3390/ma17040799

**Published:** 2024-02-07

**Authors:** Nataliya A. Sakharova, Jorge M. Antunes, André F. G. Pereira, Bruno M. Chaparro, Tomás G. Parreira, José V. Fernandes

**Affiliations:** 1 Centre for Mechanical Engineering, Materials and Processes (CEMMPRE)—Advanced Production and Intelligent Systems, Associated Laboratory (ARISE), Department of Mechanical Engineering, University of Coimbra, Rua Luís Reis Santos, Pinhal de Marrocos, 3030-788 Coimbra, Portugal; jorge.antunes@dem.uc.pt (J.M.A.); andre.pereira@uc.pt (A.F.G.P.); tomas.parreira@dem.uc.pt (T.G.P.); valdemar.fernandes@dem.uc.pt (J.V.F.); 2 Abrantes High School of Technology, Polytechnic Institute of Tomar, Quinta do Contador, Estrada da Serra, 2300-313 Tomar, Portugal; bruno.chaparro@ipt.pt

**Keywords:** aluminum nitride, gallium nitride, nanosheets, elastic moduli, modeling, numerical simulation

## Abstract

Two-dimensional (2D) nanostructures of aluminum nitride (AlN) and gallium nitride (GaN), called nanosheets, have a graphene-like atomic arrangement and represent novel materials with important upcoming applications in the fields of flexible electronics, optoelectronics, and strain engineering, among others. Knowledge of their mechanical behavior is key to the correct design and enhanced functioning of advanced 2D devices and systems based on aluminum nitride and gallium nitride nanosheets. With this background, the surface Young’s and shear moduli of AlN and GaN nanosheets over a wide range of aspect ratios were assessed using the nanoscale continuum model (NCM), also known as the molecular structural mechanics (MSM) approach. The NCM/MSM approach uses elastic beam elements to represent interatomic bonds and allows the elastic moduli of nanosheets to be evaluated in a simple way. The surface Young’s and shear moduli calculated in the current study contribute to building a reference for the evaluation of the elastic moduli of AlN and GaN nanosheets using the theoretical method. The results show that an analytical methodology can be used to assess the Young’s and shear moduli of aluminum nitride and gallium nitride nanosheets without the need for numerical simulation. An exploratory study was performed to adjust the input parameters of the numerical simulation, which led to good agreement with the results of elastic moduli available in the literature. The limitations of this method are also discussed.

## 1. Introduction

**Figure 1:**
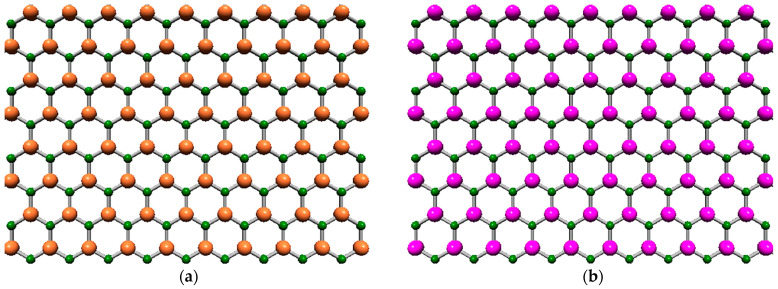
Hexagonal nanosheets: (**a**) aluminum nitride (h-AlN) and (**b**) gallium nitride (h-GaN). The N atoms are depicted in green, the Al atoms are in yellow, and the Ga atoms are in purple.

Aluminum nitride (AlN) and gallium nitride (GaN) are emerging semiconductors with a wide bandgap and possessing remarkable physical and chemical properties that place them at the forefront of diverse fields. Among these, it is worth mentioning the development of electronics, optoelectronics, electrophotonics, energy storage devices, sensors, detectors, and nanoelectromechanical systems (NEMS) [1,2]. Both AlN and GaN compounds exist in the form of two-dimensional (2D) allotropes, also called nanosheets (NSs), with a hexagonal graphene-like lattice. Such single-layer structures consist of alternating Al (Ga) and N atoms, making up a honeycomb arrangement, as shown in Figure 1. The dynamic stability and honeycomb structure with planar geometry of AlN and GaN monolayers were, for the first time, predicted by Şahin et al. [3], who used a first-principles plane-wave approach within the framework of density functional theory (DFT) calculations for strain energy and electronic structure. To this end, Zhuang et al. [4] employed DFT calculations using the projector-augmented wave (PAW) method as implemented in the Vienna ab initio simulation package (VASP).

The diatomic AlN and GaN nanostructures, presented in Figure 1, are characterized by an interatomic bond length, $a_{\mathrm{Al}(\mathrm{Ga})–N}$, whose values as reported in the literature are shown in Table 1. It is evident that there is no consensus among the research community concerning the bond length values for aluminum nitride and gallium nitride nanostructures with a graphene-like lattice.

Two-dimensional aluminum nitride and gallium nitride nanostructures exhibit enhanced properties, including mechanical properties, when compared with bulk AlN and GaN materials. Considering that nanosheets are easy to bend, they can be considered suitable candidates for flexible electronic and photoelectric nanodevices [2]. Moreover, their high porosity and mechanical stability, combined with their light weight, mean 2D AlN and GaN mono- and multilayers are promising alternatives for lithium-ion battery electrodes [2].

To facilitate understanding, the envisioned applications of aluminum nitride and gallium nitride NSs are summarized in Table 2.

The possibility of modifying the electronic (namely, band structure and direct–indirect band gap transition) [17,36,37,38,39], thermoelectric [36], and optical (such as optical spectrum shifts) [38] properties by introducing different types of deformations into 2D AlN and GaN nanostructures points to a promising future for these materials in the field of strain engineering. In addition, it has been shown that joining aluminum nitride and gallium nitride nanosheets leads to a significant increase in the thermal conductivity of the resulting heterostructures [40]. The thermal conductivity values found for these heterostructures were between those for the AlN and GaN monolayers, which contradicts the common belief that alloying results in a decrease in thermal conductivity [40].

Facing the upcoming technological demands for AlN and GaN nanosheets, substantial research efforts have been devoted to developing techniques for their synthesis and manufacturing. The preparation methods for aluminum nitride nanosheets (AlNNSs) so far comprise chemical vapor deposition (CVD), molecular beam epitaxy (MBE), physical vapor transport (PVT), and metal–organic chemical vapor deposition (MOCVD). For the first time, Zhang et al. [41] synthesized single-crystalline AlN nanosheets on Si substrates using the CVD technique. Later, Tsipas et al. [42] reported successful growth of 2D AlN with a graphene-like hexagonal lattice, resorting to plasma-assisted MBE using single-crystal Ag as a substrate. Mansurov et al. [43] grew an h-AlN layer on a Si substrate using ammonia MBE. Borisenko et al. [44] employed the plasma-assisted MBE method using graphene/silicon dioxide/silicon as a substrate to produce an AlN nanolayer. Yang et al. [45] proposed the PVT method and utilized it to produce 2D AlN with a hexagonal crystal structure in large quantities. Regarding the MOCVD technique, Wang et al. [46] first employed it to grow 2D AlN layers with a wurtzite-type structure sandwiched between graphene and Si substrates. Chen et al. [12] grew AlN nanolayers on graphene/sapphire substrates by MOCVD using trimethylaluminum (TMAl) as a precursor. Chang et al. [47] deposited 2D AlN layers on a nanopatterned sapphire substrate using MOCVD with ammonia and TMAl as precursors. The procedures used in the works of Chen et al. [12] and Chang et al. [47] make it possible to obtain high-quality AlN nanosheets that can be used to manufacture DUV-LEDs. Kakanakova-Georgieva et al. [48] grew nanometer-thick AlN on epitaxial graphene by resorting to MOCVD and using their own previous ab initio molecular dynamics (AIMD) simulations to guide the synthesis process.

Advances in the preparation of gallium aluminum nanosheets (GaNNSs) are connected with the use of electrochemical etching (ECE), UV-assisted electroless chemical etching, nitrification reactions, and graphene-assisted growth. Xiong et al. [49] prepared 2D single-crystalline GaN nanolayers using the ECE method. Recently, Zhang et al. [50] performed ECE with amino acids to obtain 2D h-GaN nanostructures. ElAfandy et al. [51] used UV-assisted chemical etching with electrolytes based on hydrofluoric acid instead of ECE to prepare 2D single-crystalline GaN nanolayers that were dislocation- and tension-free. Liu et al. [52] used a template method for the synthesis of GaN nanosheets with a hexagonal wurtzite crystal structure. The 2D γ-Ga_2_O_3_ layer, prepared by hydrothermal reaction, was used as a template for transformation into a 2D GaN nanosheet via nitridation. Recently, Zhao et al. [53] optimized the template method of Liu et al. [52] to develop a low-cost process to synthesize high-quality GaNNSs. Chen et al. [22] used CVD combined with a surface-confined nitrification reaction to grow 2D single-crystalline GaN nanosheets. Concerning graphene-assisted growth of GaNNSs, Baluchi et al. [54] synthesized, for the first time, 2D layers of GaN using epitaxial graphene by a migration-enhanced encapsulated growth (MEEG) technique. Wang et al. [55] grew 2D GaN nanosheets with a wurtzite lattice structure on a graphene/Si substrate via plasma-assisted MOCVD. Sun et al. [24] prepared 2D GaN nanosheets employing graphene oxide sheets as templates, finishing the process with a nitrification reaction. Sfuncia et al. [56], using the results of AIMD simulations to guide the MOCVD process, grew a hexagonal GaN monolayer in confinement at the graphene/SiC interface. GaNNSs synthesized by ECE techniques [49,50] are suitable components for FETs, and those obtained using graphene-assisted methods [24,54,55] are considered anode materials for Li-ion batteries and constituents of LEDs.

Moreover, ultrathin crystalline AlN/GaN nanomembranes were prepared by Mei et al. [57] on Si substrate using metal–organic vapor phase epitaxy (MOVPE). These nanomembranes are potential candidates for molecular separation and artificial blood capillaries. Deen et al. [58] grew AlN/GaN heterostructures on lattice-matched free-standing GaN substrates by RF-plasma-assisted MBE, suggesting their application as high electron mobility transistors.

To overcome the difficulties of experimental techniques for preparing 2D AlN and GaN layers, special research attention has been paid to their computational synthesis. Therefore, with the help of DFT calculations, Singh et al. [59], using the projector-augmented wave method as implemented in the PAW code VASP, determined appropriate synthesis conditions for 2D h-AlN and h-GaN and specified the most suitable substrates for their growth. Singh and Hennig [60] used the same computational approach to identify refractory materials suitable as substrates for single-layer h-GaN synthesis.

The mechanical response of AlNNSs and GaNNSs is an important issue because knowledge of it is crucial for understanding the appropriate use of these materials in flexible electronics and optoelectronics, energy storage, NEMS, and strain engineering applications. Investigations devoted to the evaluation of the mechanical properties of AlN and GaN nanosheets have been only theoretical (analytical and numerical) to date, and most of the studies used atomistic approaches comprising ab initio DFT and molecular dynamics (MD) calculations. The ab initio method requires only fundamental physical constants as input, making it appropriate for a small number of atoms as it consumes large amounts of computational resources. Jafaria et al. [61], Peng et al. [62], Kourra et al. [63], and Lv et al. [64] employed ab initio DFT calculations to evaluate the elastic properties of ANNSs. Tuoc et al. [65] and Fabris et al. [66] used the same method to study the elastic behavior of GaNNSs. Regarding the evaluation of the elastic properties of aluminum nitride and gallium nitride NSs, Ahangari et al. [6], Luo et al. [67], Faraji et al. [68], and Ye and Peng [69] also used ab initio DFT calculations. In general, ab initio DFT methods provide more accurate results than MD, which is more efficient when large atomic arrangements are considered and whose outcomes are, to a great extent, influenced by the potential functions chosen for describing the interactions between Al (Ga) and N atoms of the diatomic nanostructure. Rouhi et al. [70] performed MD simulations with the Tersoff–Brenner (TB) potential function to study the mechanical properties of GaNNSs. Singh et al. [71] used the TB potential to model the interactions between Al and N atoms in their molecular statics (MS) simulation study on determining the elastic constants of AlNNSs. Le [8] investigated the tensile properties of AlN and GaN nanosheets employing MD simulation with Tersoff potentials. Sarma et al. [72] studied the mechanical behavior of GaNNSs using MD simulations with the Stillinger–Weber (SW) potential.

The atomistic approaches, being computationally time-consuming, have been progressively substituted by the nanoscale continuum modeling (NCM) approach, which has already been successfully used to model the mechanical behavior of 2D nanostructures with a graphene-like lattice (see, for example, [73,74,75]). The NCM approach, also called molecular structural mechanics (MSM), uses the connection between the molecular structure of the NS and solid mechanics such that the bonds between Al (Ga) and N atoms are modeled as elastic elements, most commonly beams or springs. Despite the simpler mathematical formulation compared to the atomistic approaches, as well as the simplicity and speed of implementation, the NCM/MSM method has not been widely employed to study the mechanical behavior of aluminum nitride and gallium nitride NSs. Under the NCM/MSM approach, Le [76] derived a closed-form expression to assess the Young’s modulus of AlNNSs. Using the same modeling method, Ben et al. [2] calculated the maximum stress and strain in tension of AlNNSs and GaNNSs, resorting to the respective closed-form solutions. Giannopoulos et al. [77] represented interatomic bonds in GaNNSs as spring elements within the NCM/MSM approach and studied the tensile behavior of NSs. It is worth noting that existing analytical and numerical studies generally focus on square nanosheets of either AlN or GaN and less frequently on both compounds simultaneously, thus lacking systematic investigation. To our knowledge, only Giannopoulos et al. [77] and Rouhi et al. [70] studied the effect of nanosheet size and aspect ratio, respectively, on the tensile behavior of GaNNSs. Moreover, a certain discrepancy is observed in the elastic properties of NSs reported to date.

In view of the promising applications envisaged for 2D AlN and GaN structures, it is crucial to develop a straightforward methodology that allows the reliable characterization of their elastic properties. In this context, the goal of the present study was to evaluate the Young’s and shear moduli of single-layer aluminum nitride and gallium nitride nanosheets, varying their aspect ratio to cover a large number of NS sizes and shapes (from square to rectangular). For this purpose, the Al–N and Ga–N interatomic bonds were simulated as equivalent beams within the NCM/MSM approach, and three-dimensional (3D) finite element (FE) models of AlNNSs and GaNNSs were built. The mechanical behavior of AlNNSs and GaNNSs in a wide range of aspect ratios was investigated under loading conditions applied in numerical in-plane tensile and shear tests. As a result, for the first time, an analytical methodology was established that allows the calculation of AlNNS and GaNNS elastic moduli without the need for numerical simulation. In this way, the present work is a systematic study, the results of which contribute to the design and manufacture of flexible electronic and optoelectronic nanodevices and are useful for upcoming developments in strain engineering.

## 2. Materials and Methods

### 2.1. Modeling and Numerical Simulation of the Elastic Behavior of AlN and GaN Nanosheets

#### 2.1.1. Geometrical Characteristics of AlNNSs and GaNNSs

Single-layer AlNNSs and GaNNSs with different aspect ratios, as shown in Table 3, were studied. By changing the aspect ratio of the nanosheets so that their size varied in the range from ≈3 × 3 nm^2^ to ≈15 × 15 nm^2^, it was possible to obtain 5 square NSs and 20 rectangular NSs of diverse configurations for each compound. The geometrical properties of AlN and GaN nanosheets were systematized such that, in each group, the vertical lateral length, $L_{y}$, would always be the same and the horizontal lateral length, $L_{x}$, was variable.

The finite element (FE) meshes of AlNNSs and GaNNSs were taken as the program database files with the Nanotube Modeler^®^ software (version 1.8.0, ©JCrystalSoft, http://www.jcrystal.com, 4 January 2024). Afterwards, these program database files were converted to a format usable in commercial codes for finite element analysis (FEA) using the in-house application InterfaceNanosheets.NS [73].

**Figure 2:**
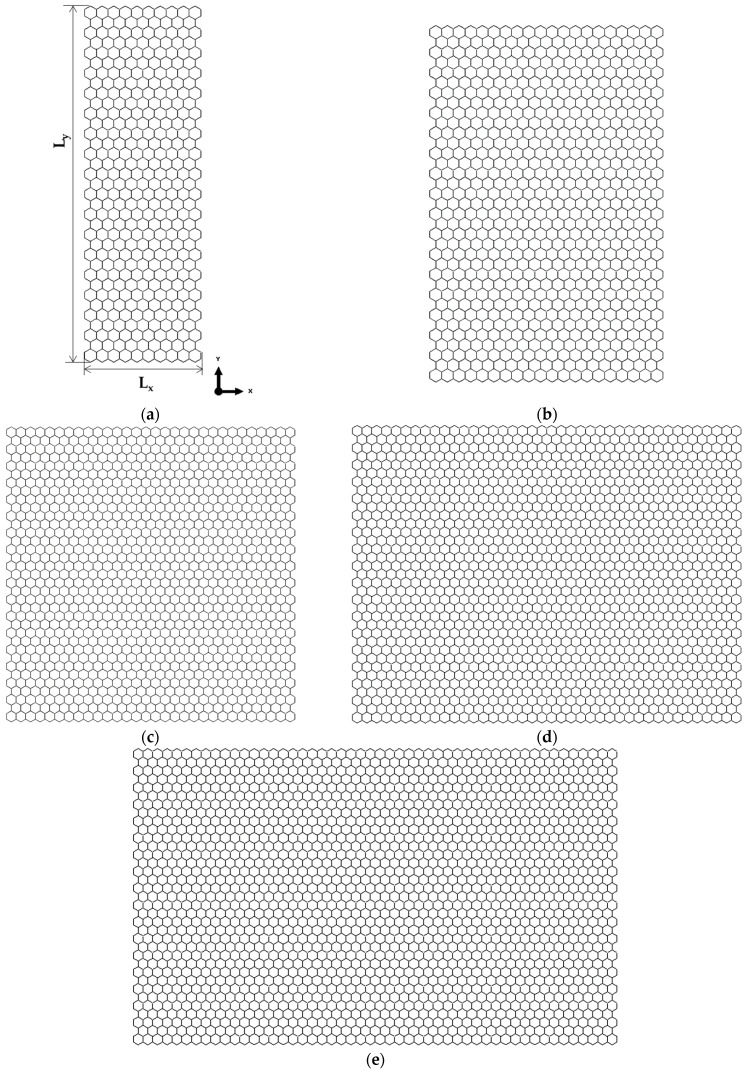
FE meshes of AlNNSs with $L_{y}$ = 9.70 nm and $L_{x}/L_{y}$ equal to (**a**) 0.3, (**b**) 0.7, (**c**) 1, (**d**) 1.3, and (**e**) 1.6.

**Figure 3:**
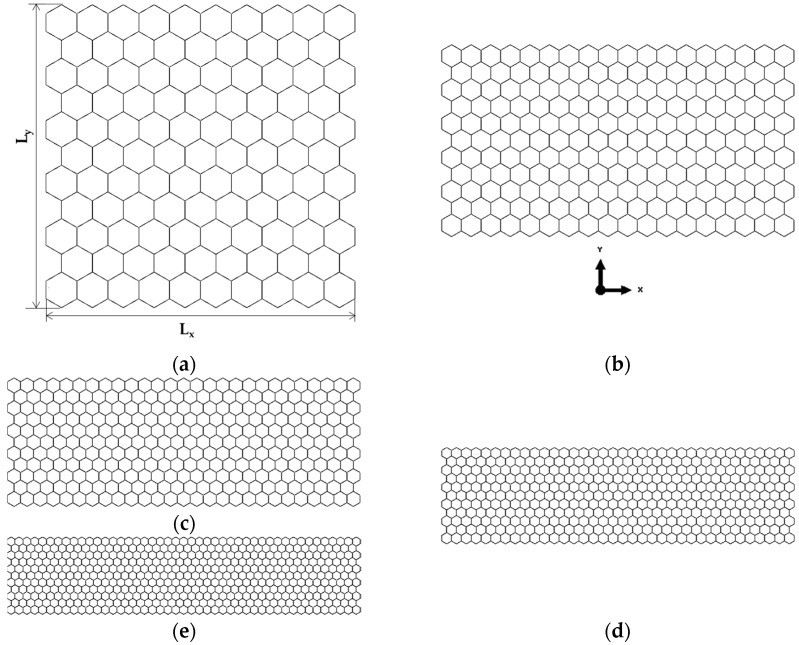
FE meshes of GaNNSs with $L_{y}$ = 3.32 nm and $L_{x}/L_{y}$ equal to (**a**) 1, (**b**) 1.8, (**c**) 2.7, (**d**) 3.7, and (**e**) 4.6.

Examples of FE meshes for AlN nanosheets with a fixed NS vertical lateral length, $L_{y}$, of 9.70 nm and variable NS horizontal lateral length, $L_{x}$, in the range of 3.17 to 15.83 nm and for GaNNSs with $L_{y}$ of 3.32 nm and $L_{x}$ ranging from 3.38 to 15.17 nm are shown in Figure 2 and Figure 3, respectively.

It is possible to separate the geometrical configuration of the nanosheets in relation to their aspect ratio, $L_{x}$/$L_{y}$. AlNNSs and GaNNSs with $L_{x}$/$L_{y}$ < 1 are rectangular, with the vertical NS side greater than the horizontal (Figure 2a,b). When $L_{x}$/$L_{y}$ > 1, AlNNSs and GaNNSs are rectangular, with the horizontal side greater than the vertical (Figure 2d,e and Figure 3b–d). Finally, in square nanosheets, $L_{x}$/$L_{y}$ = 1 (see Figure 2c and Figure 3a).

#### 2.1.2. Molecular Mechanics of AlNNSs and GaNNSs and Equivalent Continuum Properties of Al–N and Ga–N Bonds

In the current study, the NCM/MSM method was employed to evaluate the elastic moduli of AlNNSs and GaNNSs. This modeling approach is based on the linkage between the molecular structure of the hexagonal diatomic lattice and the equivalent continuum structure. Such a connection is accomplished by replacing the Al (Ga)–N bonds with equivalent beam elements such that the resulting continuum structure of the NSs consists of elastic beams.

Potential energies of the interatomic bonded interactions of the molecular structure related to bond stretching (U_r_), bond bending (U_θ_), and bond torsion (U_τ_) are given by the following expressions [78]:

(1) $$U_{r}=\frac{1}{2}k_{r}{\left(\Delta r\right)}^{2},$$

(2) $$U_{\theta }=\frac{1}{2}k_{\theta }{\left(\Delta \theta \right)}^{2},$$

(3) $$U_{\tau }=\frac{1}{2}k_{\tau }{\left(\Delta \phi \right)}^{2}.$$

Here, $k_{r}$, $k_{\theta }$, and $k_{\tau }$ are the bond stretching, bond bending, and torsional resistance force constants, respectively; Δr, Δθ, and Δϕ are the bond stretching increment, the bending bond angle variation, and the twist bond angle variation, respectively.

Regarding the equivalent continuum structure, the elastic strain energies associated with the axial (U_A_), bending (U_B_), and torsional (U_T_) strains of the constituting equivalent beams are expressed as follows:

(4) $$U_{A}=\frac{1}{2}\frac{E_{b}A_{b}}{l}{\left(\Delta l\right)}^{2},$$

(5) $$U_{B}=\frac{1}{2}\frac{E_{b}I_{b}}{l}{\left(2\omega \right)}^{2},$$

(6) $$U_{T}=\frac{1}{2}\frac{G_{b}J_{b}}{l}{\left(\Delta \vartheta \right)}^{2}.$$

Here, $E_{b}A_{b}$, $E_{b}I_{b}$, and $G_{b}J_{b}$ are the tensile, bending, and torsional rigidities of the beam with length *l*, respectively; Δ*l* is the axial displacement of the beam in tension; ω is the rotational angle of the beam ends during bending; and Δϑ is the relative rotation between the beam ends in torsion.

The relationships between the beam $E_{b}A_{b}$, $E_{b}I_{b}$, and $G_{b}J_{b}$ rigidities and the $k_{r}$, $k_{\theta }$, and $k_{\tau }$ force constants are obtained by equating $U_{r}=U_{A}$, $U_{\theta }=U_{B}$, and $U_{\tau }=U_{T}$ using expressions (1)–(3) and (4)–(6), respectively [79]:

(7) $$E_{b}A_{b}=lk_{r},\;E_{b}I_{b}=lk_{\theta },\;G_{b}J_{b}=lk_{\tau }.$$

Here, $A_{b}=\pi d^{2}/4$ is the cross-sectional area, $I_{b}=\pi d^{4}/64$ is the moment of inertia, and $J_{b}=\pi d^{4}/32$ is the polar moment of inertia of a beam with a circular cross-section and diameter d.

Equation (7) is the basis for the analysis of the mechanical response of AlNNSs and GaNNSs being used to calculate the input parameters for the numerical simulation, knowing the values of the force constants $k_{r}$, $k_{\theta }$, and $k_{\tau }$.

In the present work, the method based on DFT calculations in combination with analytical relationships for the surface Young’s modulus, $E_{s}$, and the Poisson’s ratio, ν, originating from molecular mechanics (MM) was used to assess $k_{r}$ and $k_{\theta }$ force field constants. To this end, the following expressions were used [73]:

(8) $$k_{r}=\frac{9E_{s}}{\sqrt{3}\left(1-\nu \right)},$$

(9) $$k_{\theta }=\frac{E_{s}a_{\mathrm{Al}(\mathrm{Ga})–N}^{2}}{2\sqrt{3}\left(1+3\nu \right)},$$

where $a_{\mathrm{Al}(\mathrm{Ga})–N}$ is the length of the Al–N (Ga–N) bond, which is equal to the beam length, *l*, in the present model. The values of $E_{s}$ and ν used in Equations (8) and (9) were taken from the results of the DFT calculations by Şahin et al. [3], as shown in Table 4, together with the bond lengths, $a_{\mathrm{Al}(\mathrm{Ga})–N}$.

The torsion resistance constant, $k_{\tau }$, was obtained using the DREIDING force field [80], where the torsional properties of the diatomic nanostructure are evaluated by uniquely taking into account the hybridization of the atoms. The values of $k_{r}$, $k_{\theta }$, and $k_{\tau }$ used are also shown in Table 4.

The values of the force field constants and the bond lengths, given in Table 4, allowed the calculation of the geometrical and elastic properties of the beams (input values for the numerical simulation) shown in Table 5, together with their respective formulations.

#### 2.1.3. FEA and Calculation of Young’s and Shear Moduli of AlN and GaN Nanosheets

**Figure 4:**
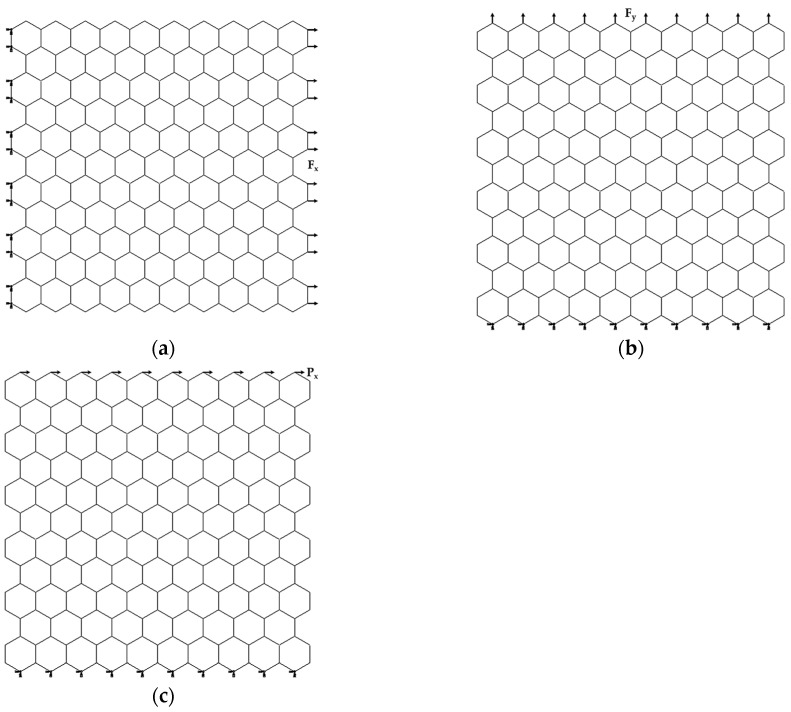
Schematic representation of the loading and boundary conditions for AlNNSs of 3.17 × 3.11 nm^2^ (see Table 3): (**a**) tensile loading in the horizontal (zigzag) direction, (**b**) tensile loading in the vertical (armchair) direction, (**c**) in-plane shear loading in the horizontal direction.

The mechanical behavior of AlNNSs and GaNNSs was investigated under numerical tensile and in-plane shear tests using the ABAQUS^®^ FE code (Abaqus 2020, Dassault Systèmes^®^, Vélizy-Villacoublay, France). Figure 4 shows the three studied loading cases with the respective boundary conditions for a square AlN nanosheet as an example.

In the first loading case, shown in Figure 4a, the nodes on the left side of the nanosheet were fixed, and an axial tensile force, $F_{x}$, was applied to the opposite side. In the second case, represented in Figure 4b, the bottom edge nodes were fixed, while an axial force, $F_{y}$, was applied to the nodes of the NS upper side. The abovementioned loading conditions meant that two tensile configurations of AlNNSs and GaNNSs, zigzag and armchair, respectively, were considered. In the third loading case (Figure 4c), the boundary conditions were the same as in the second case, and a shear force, $P_{x}$, was applied to the upper side nodes.

To calculate the Young’s modulus along the x-axis, $E_{x}$, the NS axial displacement, $u_{x}$, (corresponding to elongation in the x-direction) under the axial tensile load, $F_{x}$, was taken from FEA (see Figure 4a). Consequently, $E_{x}$ was evaluated as follows [74]:

(10) $$E_{x}=\frac{F_{x}L_{x}}{u_{x}L_{y}t_{n}},$$

where $L_{x}$ and $L_{y}$ are the NS side lengths (see Figure 2 and Figure 3), and $t_{n}$ is the nanosheet thickness.

The axial displacement of the nanosheet in the y-direction, $v_{y}$, under the applied load $F_{y}$, was taken from the FEA (Figure 4b). The Young’s modulus along the y-axis, $E_{y}$, is given by the following expression [74]:

(11) $$E_{y}=\frac{F_{y}L_{y}}{v_{y}L_{x}t_{n}}.$$

To calculate the shear strain, $\gamma_{\mathrm{xy}}$, the NS displacement in the x-direction, $r_{x}$, under the in-plane shear load $P_{x}$, was taken from the FEA (Figure 4c). Therefore, the NS shear modulus, $G_{\mathrm{xy}}$, can be evaluated as follows [74]:

(12) $$G_{\mathrm{xy}}=\frac{P_{x}}{\gamma_{\mathrm{xy}}L_{x}t_{n}},\;\gamma_{\mathrm{xy}}=tan\frac{r_{x}}{L_{y}}.$$

The displacement $r_{x}$ was measured in the central part of the NS to avoid influence at the nodes, where boundary and loading conditions were applied.

Given the lack of knowledge of the $t_{n}$ value for AlNNSs and GaNNSs, the alternative to $E_{x}$, $E_{y}$, and $G_{\mathrm{xy}}$ is to calculate the surface Young’s and shear moduli, $E_{sx}$, $E_{\mathrm{sy}}$, and $G_{\mathrm{sxy}}$ (the product of the respective elastic modulus by the nanosheet thickness). For this, Equations (10)–(12) are written as follows:

(13) $${E_{sx}=E}_{x}t_{n}=\frac{F_{x}L_{x}}{{u_{x}L}_{y}},$$

(14) $$E_{\mathrm{sy}}=E_{y}t_{n}=\frac{F_{y}L_{y}}{v_{y}L_{x}},$$

(15) $$G_{\mathrm{sxy}}=G_{\mathrm{xy}}t_{n}=\frac{P_{x}}{\gamma_{\mathrm{xy}}L_{x}}.$$

## 3. Results and Discussion

### 3.1. Parametric Study on Surface Elastic Moduli of AlN and GaN Nanosheets

#### 3.1.1. Size Effect on Surface Young’s and Shear Moduli of Square AlNNSs and GaNNSs

**Figure 5:**
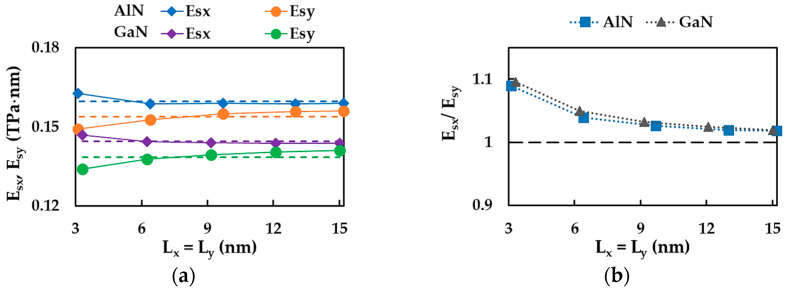
(**a**) Surface Young’s moduli, $E_{\mathrm{sx}}$ (zigzag) and $E_{\mathrm{sy}}$ (armchair) of square AlNNSs and GaNNSs (Table 3); the dashed lines represent the average values of $E_{\mathrm{sx}}$ and $E_{\mathrm{sy}}$. (**b**) Evolutions of the ratio between the surface Young’s moduli in the zigzag and armchair directions, $E_{\mathrm{sx}}/E_{\mathrm{sy}}$, for the same NSs as in (**a**); the dashed line corresponds to $E_{\mathrm{sx}}/E_{\mathrm{sy}}$ = 1 (isotropy).

Figure 5a shows the surface Young’s moduli for the zigzag configuration (along the x-direction), $E_{\mathrm{sx}}$, and armchair configuration (along the y-direction), $E_{\mathrm{sy}}$, calculated by Equations (13) and (14), respectively, from the tensile simulations of AlN and GaN square nanosheets of five different sizes (see Table 3). For AlN and GaN compounds, the surface Young’s modulus along the x-direction, $E_{\mathrm{sx}}$, was almost constant for all sizes of NSs studied, except for the nanosheet with the smallest side length, $L_{x}$ ≈ $L_{y}$ ≈ 3 nm, for which the value of $E_{\mathrm{sx}}$ increased. The surface Young’s modulus along the y-direction, $E_{\mathrm{sy}}$, increased slightly from the NS of the smallest size (≈3 × 3 nm^2^) to that of the largest size studied (with a side length $L_{x}$ ≈ $L_{y}$ ≈ 15 nm) by 4.6% and 5.2% for AlN and GaN nanosheets, respectively. The average values of the surface Young’s moduli, $E_{\mathrm{sx}}$ and $E_{\mathrm{sy}}$, of both nanosheets are represented in Figure 5a by dashed lines. These average values were, on the one hand, $E_{\mathrm{sx}}$ = 0.160 and 0.144 TPa·nm and, on the other hand, $E_{\mathrm{sy}}$ = 0.154 and 0.138 TPa·nm for AlNNSs and GaNNSs, respectively. Thus, for square AlNNSs, the $E_{\mathrm{sx},y}$ average values were about 10.5% higher than those of GaNNSs. The lower values of $E_{\mathrm{sx},y}$ moduli calculated for GaNNSs can probably be justified by the longer length of the Ga–N bond, $a_{\mathrm{Ga}–N}$ = 0.185 nm, when compared with the length of the Al–N bond, which was $a_{\mathrm{Al}–N}$ = 0.179 nm.

As can be seen in Figure 5b, the surface Young’s modulus of both AlNNSs and GaNNSs was greater in the zigzag direction than in the armchair direction, $E_{\mathrm{sx}}$ > $E_{\mathrm{sy}}$, which suggests that the square aluminum nitride and gallium nitride NSs are not transversely isotropic. In this way, the ratio between the surface Young’s moduli for the zigzag and armchair configurations, $E_{\mathrm{sx}}/E_{\mathrm{sy}}$, can quantify the anisotropic behavior of NSs. The $E_{\mathrm{sx}}/E_{\mathrm{sy}}$ ratio decreased from nearly 1.093 to 1.019 when the NS size increased, irrespective of whether the compound was AlN or GaN (see Figure 5b). The average values of the ratio were $E_{\mathrm{sx}}/E_{\mathrm{sy}}$ ≈ 1.038 and 1.043 for AlNNSs and GaNNSs, respectively. A mild anisotropy with $E_{\mathrm{sx}}/E_{\mathrm{sy}}$ = 1.031 was previously reported by Sakharova et al. [73] for boron nitride nanosheets (BNNSs), which are representatives of the 13th group, i.e., nitride nanostructures as well as AlNNSs and GaNNSs. In that study, the anisotropic behavior of NSs was explained by the different stresses needed for stretching the hexagonal lattice in the zigzag and armchair directions when the respective axial force was applied due to the atom’s arrangement. With regard to the $E_{\mathrm{sx}}/E_{\mathrm{sy}}$ ratio, it can be concluded that its value increases with an increase in the bond length. Indeed, the converged average value of the $E_{\mathrm{sx}}/E_{\mathrm{sy}}$ ratio increased from 1.019 (BN) [73] to 1.026 (AlN) and then to 1.031 (GaN) with an increase in the value of $a_{B–N}$ = 0.145 nm < $a_{\mathrm{Al}–N}$ = 0.179 nm < $a_{\mathrm{Ga}–N}$ = 0.185 nm [3].

**Figure 6:**
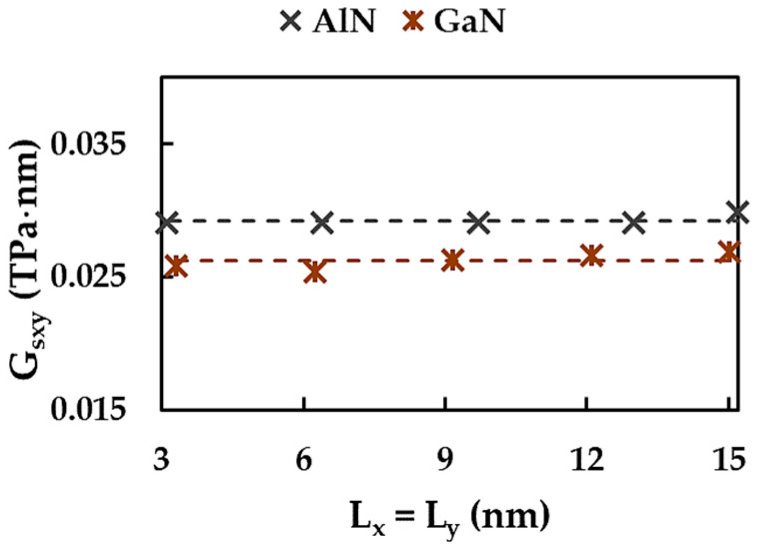
Surface shear modulus, $G_{\mathrm{sxy}}$, of the square AlNNSs and GaNNSs. The dashed lines represent the average values of $G_{\mathrm{sxy}}$.

Figure 6 shows the surface shear modulus, $G_{\mathrm{sxy}}$, for the square AlNNSs and GaNNSs, calculated by Equation (15), from the shear test simulations. The $G_{\mathrm{sxy}}$ values were nearly constant for all square AlN and GaN nanosheets studied, although the shear modulus values obtained for GaNNSs showed greater scattering.

The average surface shear modulus of AlNNSs, $G_{\mathrm{sxy}}$ = 0.029 TPa·nm, was about 12% higher than that of GaNNSs, which was $G_{\mathrm{sxy}}$ = 0.026 TPa·nm.

As was established previously, among 2D materials in the 13th group (nitride compounds), BN nanosheets exhibited superior mechanical properties, close to those of graphene [73,81]. The surface Young’s and shear moduli of the BNNSs, $E_{\mathrm{sx}}$ = 0.334 TPa·nm, $E_{\mathrm{sy}}$ = 0.324 TPa·nm, and $G_{\mathrm{sxy}}$ = 0.065 TPa·nm [71], were nearly 2 and 2.5 times greater than the $E_{\mathrm{sx},y}$ and $G_{\mathrm{sxy}}$ values calculated for AlNNSs and GaNNSs, respectively. This should be taken into account in the assembly of heterostructures involving 2D nitrides and in the design of nanodevices, where the mechanical strength of the components is important.

#### 3.1.2. Influence of the Aspect Ratio on the Surface Elastic Moduli of AlNNSs and GaNNSs

Next, the influence of the aspect ratio on the surface elastic moduli of aluminum nitride and gallium nitride NSs was analyzed. To this end, the approach used was similar to that proposed by Georgantzinos et al. [75] for the study of the elastic behavior of BNNSs.

**Figure 7:**
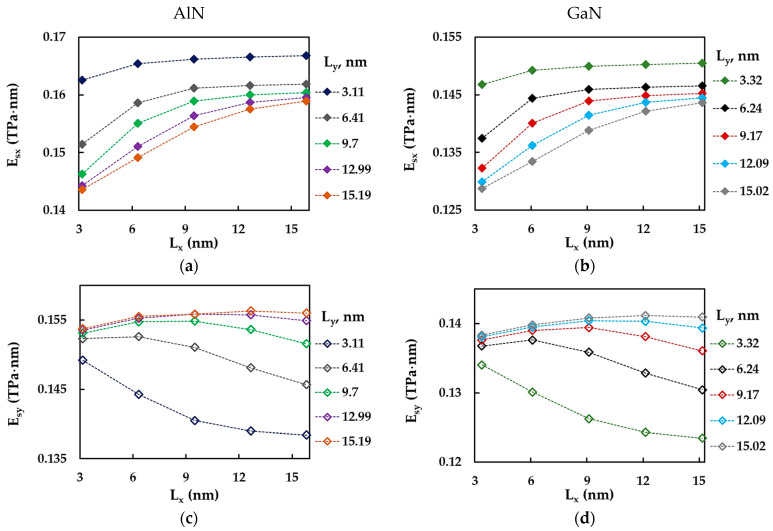
Evolutions of the surface Young’s modulus for the zigzag configuration, $E_{\mathrm{sx}}$, for (**a**) AlNNSs and (**b**) GaNNSs and also for the armchair configuration, $E_{\mathrm{sy}}$, for (**c**) AlNNSs and (**d**) GaNNSs as a function of the NS side length, $L_{x}$.

To understand the effect of the aspect ratio on AlNNS and GaNNS surface Young’s moduli, the evolutions of the Young’s moduli along the x-direction, $E_{\mathrm{sx}}$, and along the y-direction, $E_{\mathrm{sy}}$, were plotted as a function of the NS horizontal side length, $L_{x}$, as shown in Figure 7. Five sets of nanosheets, each with the same NS vertical lateral length, $L_{y}$, as presented in Table 3, were considered for AlNNSs and GaNNSs.

The trend in the evolution of the surface Young’s modulus in the zigzag direction, which can be observed in Figure 7a,b, showed that $E_{\mathrm{sx}}$ increased with increasing NS side length, $L_{x}$. For ANNSs and GaNNSs with the aspect ratio $L_{x}$/$L_{y}$ < 1, the increasing rate was considerable until $E_{\mathrm{sx}}$ attained the value for the square NS configuration ($L_{x}$/$L_{y}$ = 1). Afterwards, with further increase in $L_{x}$, the growth of the surface Young’s modulus slowed down. This last domain of the $E_{\mathrm{sx}}$ evolution corresponded to nanosheets with $L_{x}$/$L_{y}$ > 1, which were larger in width than in height. With regard to $E_{\mathrm{sx}}$ values, the greater the vertical side length, $L_{y}$, the lower the surface Young’s modulus along the x-direction. To facilitate understanding, the $E_{\mathrm{sx}}$ results from Figure 7a,b are summarized in Table A1 (Appendix A).

The evolutions of the surface Young’s modulus in the armchair direction, $E_{\mathrm{sy}}$, as a function of the NS side length, $L_{x}$, shown in Figure 7c,d for AlNNSs and GaNNSs, respectively, can also be analyzed in terms of the NS aspect ratio, $L_{x}$/$L_{y}$, as for the case of the $E_{\mathrm{sx}}$ modulus. For $L_{x}$/$L_{y}$ < 1 (the case of NSs shown in Figure 2a,b), $E_{\mathrm{sy}}$ slightly increased with increasing $L_{x}$ length until the aspect ratio was $L_{x}$/$L_{y}$ = 1 (square NSs). Then, when $L_{x}$ further increased, which corresponded to nanosheets with $L_{x}$/$L_{y}$ > 1, the $E_{\mathrm{sy}}$ modulus started to decrease. The more significant the decreasing rate, the smaller the value of $L_{y}$ (see Figure 7c,d). Also, lower $E_{\mathrm{sy}}$ values of AlNNSs and GaNNSs were observed when the vertical side length, $L_{y}$, decreased. The $E_{\mathrm{sy}}$ results from Figure 7c,d, are shown in Table A2 (Appendix A).

**Figure 8:**
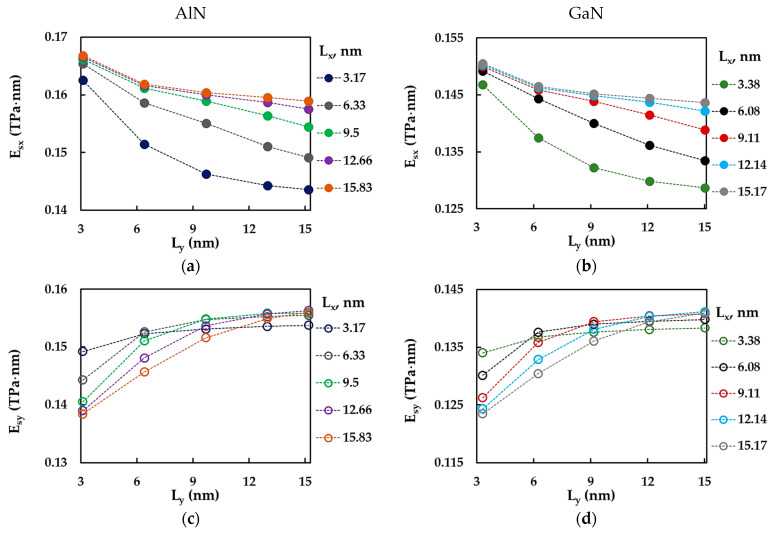
Evolutions of the surface Young’s modulus for the zigzag configuration, $E_{\mathrm{sx}}$, for (**a**) AlNNSs and (**b**) GaNNSs and also for the armchair configuration, $E_{\mathrm{sy}}$, for (**c**) AlNNSs and (**d**) GaNNSs as a function of the NS side length, $L_{y}$.

To complete the analysis of the effect of the NS aspect ratio on the $E_{\mathrm{sx}}$ and $E_{\mathrm{sy}}$ moduli, these were plotted in Figure 8 as a function of the vertical NS side length, $L_{y}$. Similar to Figure 7, Figure 8 shows the $E_{\mathrm{sx},y}$ results for the five groups of NSs, each with the same horizontal side length, $L_{x}$, for AlNNSs and GaNNSs. The Young’s modulus in the x-direction (zigzag), $E_{\mathrm{sx}}$, decreased as the vertical side length, $L_{y}$, increased. This decrease became less significant as the length of the horizontal NS side, $L_{x}$, increased (Figure 8a,b). The Young’s modulus in the y-direction (armchair), $E_{\mathrm{sy}}$, increased with increasing vertical side length, $L_{y}$, and then tended to reach a stable value with further increase in $L_{y}$. This trend was more pronounced for AlNNSs and GaNNSs with a smaller horizontal side, $L_{x}$, and an aspect ratio $L_{x}$/$L_{y}$ > 1 (see Figure 8c,d).

**Figure 9:**
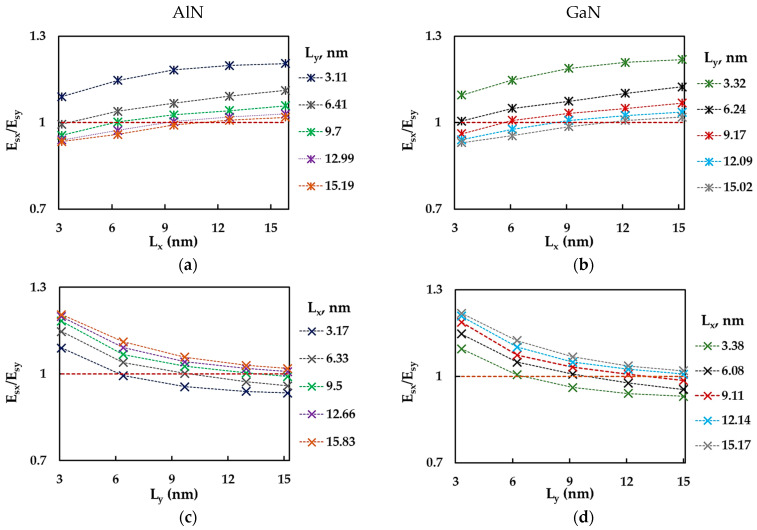
Evolutions of the $E_{\mathrm{sx}}/E_{\mathrm{sy}}$ ratio as a function of (**a**,**b**) the horizontal side length, $L_{x}$, and (**c**,**d**) the vertical side length, $L_{y}$, for (**a**,**c**) AlN and (**b**,**d**) GaN nanosheets. The horizontal dashed lines correspond to $E_{\mathrm{sx}}/E_{\mathrm{sy}}$ = 1 (nanosheet isotropy).

Figure 9 shows the evolutions of the ratio between the surface Young’s moduli for the zigzag and armchair configurations of AlNNSs and GaNNSs, $E_{\mathrm{sx}}/E_{\mathrm{sy}}$, as a function of the horizontal, $L_{x}$, and vertical, $L_{y}$, side lengths of the NS. The $E_{\mathrm{sx}}/E_{\mathrm{sy}}$ ratio increased with increasing $L_{x}$ (Figure 9a,b). For NSs with the relationship between side lengths $L_{x}$/$L_{y}$ < 1, the value of the ratio between the surface Young’s moduli was less than or close to 1 ($E_{\mathrm{sx}}/E_{\mathrm{sy}}$ = 1 corresponds to the case of NS isotropy). Also, it can be observed in Figure 9a,b that the bigger the vertical NS side, $L_{y}$, the lower the $E_{\mathrm{sx}}/E_{\mathrm{sy}}$ ratio. The evolutions of the $E_{\mathrm{sx}}/E_{\mathrm{sy}}$ ratio as a function of the vertical NS length, $L_{y}$, had the opposite trend, with $E_{\mathrm{sx}}/E_{\mathrm{sy}}$ decreasing when the length $L_{y}$ increased and $E_{\mathrm{sx}}/E_{\mathrm{sy}}$ ≲ 1 for nanosheets with the largest horizontal side length, $L_{x}$ (see Figure 9c,d). The $E_{\mathrm{sx}}/E_{\mathrm{sy}}$ results from Figure 9 are summarized in Table A3 (Appendix A).

**Figure 10:**
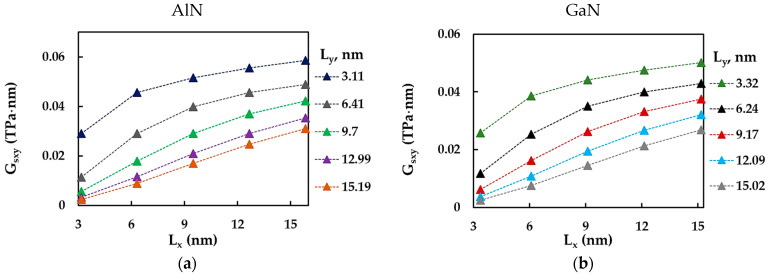

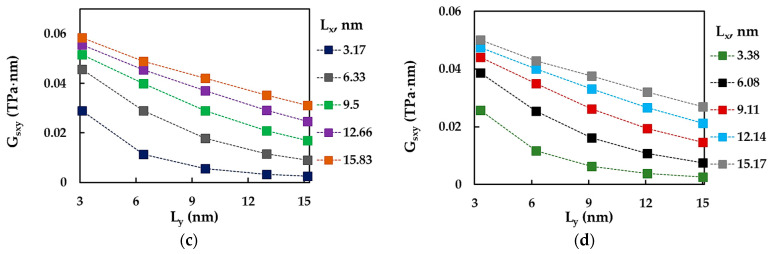
Evolutions of the surface shear modulus, $G_{\mathrm{sxy}}$, as a function of (**a**,**b**) the horizontal side length, $L_{x}$, and (**c**,**d**) the vertical side length, $L_{y}$, for (**a**,**c**) AlN and (**b**,**d**) GaN nanosheets.

The evolutions of the surface shear modulus, $G_{\mathrm{sxy}}$, of AlNNSs and GaNNSs with the horizontal, $L_{x}$, and vertical, $L_{y}$, side lengths are analyzed in Figure 10a,b and Figure 10c,d, respectively.

The surface shear modulus, $G_{\mathrm{sxy}}$, of AlNNSs and GaNNSs increased with increasing $L_{x}$ side length and, consequently, the NS aspect ratio, $L_{x}$/$L_{y}$. The $G_{\mathrm{sxy}}$ values were lower for NSs with bigger $L_{y}$ length. For the $G_{\mathrm{sxy}}$ evolutions as a function of the vertical side length, $L_{y}$, the inverse trend was observed, i.e., $G_{\mathrm{sxy}}$ decreased with increasing $L_{y}$. For $L_{x,y}$ ≈ 12 and 15 nm, the evolutions of the surface shear modulus followed an almost linear trend. The results shown in Figure 10 are summarized in Table A4 (Appendix A).

### 3.2. Development of an Analytical Approach for Determining the Surface Elastic Moduli of AlNNSs and GaNNSs

**Figure 11:**
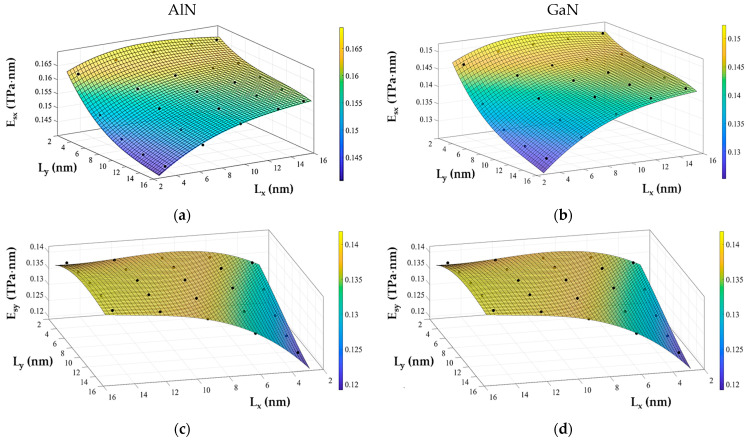

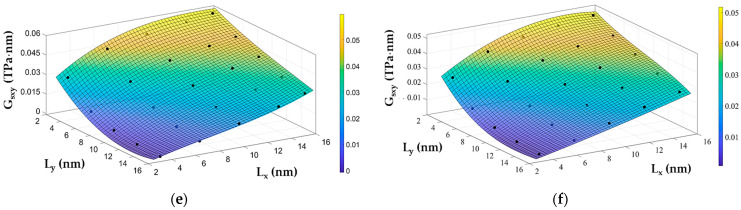
Evolutions of (**a**,**b**) the surface Young’s modulus (zigzag), $E_{\mathrm{sx}}$, (**c**,**d**) the surface Young’s modulus (armchair), $E_{\mathrm{sy}}$, and (**e**,**f**) the surface shear modulus, $G_{\mathrm{sxy}}$, as a function the horizontal, $L_{x}$, and vertical, $L_{y}$, side lengths for (**a**,**c**,**e**) AlN and (**b**,**d**,**f**) GaN nanosheets.

The surface Young’s and shear moduli evolutions discussed in Section 3.1.2, namely, the influence of the aspect ratio on the surface elastic moduli of AlNNSs and GaNNSs, is represented in the form of 3D graphs in Figure 11, where each elastic modulus, $E_{\mathrm{sx}}$, $E_{\mathrm{sy}}$, and $G_{\mathrm{sxy}}$, is plotted as a function of both NS side lengths, $L_{x}$ and $L_{y}$.

Each of the surfaces obtained in Figure 11 can be well fitted by a third-order polynomial function as follows:

(16) $$E_{\mathrm{sx},y}\left(G_{\mathrm{sxy}}\right)=P_{00}+P_{10}L_{y}+P_{01}L_{x}+P_{20}L_{y}^{2}+P_{11}L_{y}L_{x}+P_{02}L_{x}^{2}+P_{30}L_{y}^{3}+P_{21}L_{y}^{2}L_{x}+P_{12}L_{y}L_{x}^{2}+P_{03}L_{x}^{3}$$

where $P_{00}$, $P_{10}$, $P_{01}$, $P_{20}$, $P_{11}$, $P_{02}$, $P_{30}$, $P_{21}$, $P_{12}$, and $P_{03}$ are empirically obtained fitting coefficients that, together with the R-square values, are shown in Table A5 and Table A6 (Appendix B) for AlNNSs and GaNNSs, respectively.

Knowing the size of the nanosheet and the values of the coefficients in Equation (16), it is possible to calculate the surface Young’s, $E_{\mathrm{sx}}$ and $E_{\mathrm{sy}}$, and shear, $G_{\mathrm{sxy}}$, moduli of AlNNSs and GaNNSs for sizes in the range of ≈ 3 × 3 to 15 × 15 nm^2^ without resorting to numerical simulation. To validate the analytical expressions proposed, two sets of AlN and GaN nanosheets, one for each compound, whose geometrical properties are shown in Table 6, were used.

Table 7 summarizes the average differences between the values of the $E_{\mathrm{sx}}$, $E_{\mathrm{sy}}$, and $G_{\mathrm{sxy}}$ moduli calculated with the aid of the respective parameters in Equation (16) (see Table A5 and Table A6 (Appendix B)) for AlNNSs and GaNNSs and the corresponding values acquired in the numerical simulation.

It can be concluded that the analytical expressions based on the respective fitting coefficients in Equation (16) allowed a precise assessment of the surface elastic moduli, $E_{\mathrm{sx},y}$ and $G_{\mathrm{sxy}}$, of AlN and GaN nanosheets. For GaNNSs, the analytical results were less accurate for the nanosheets situated at the edge limit of the surfaces, as depicted in Figure 11b,d,f.

To the best of our knowledge, an accurate methodology for determining the elastic moduli of AlNNSs and GaNNSs without the need for numerical simulation is proposed here for the first time. Georgantzinos et al. [75] derived analytical expressions for evaluation of the elastic constants of BNNSs based on a fitting procedure different from that suggested in the current study.

### 3.3. Validation of the Current Results of Surface Elastic Moduli for AlNNSs and GaNNSs

#### 3.3.1. Comparison with Literature Results

**Figure 12:**
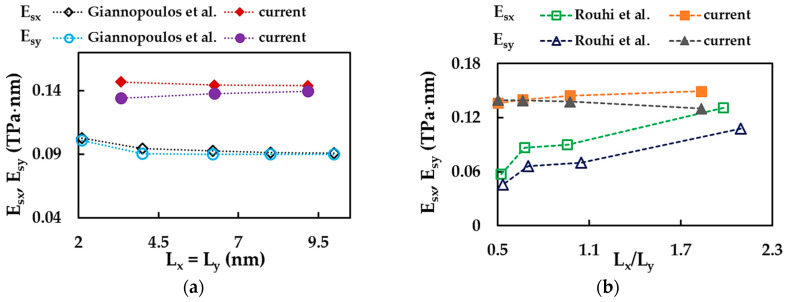
Evolutions of the surface Young’s moduli, $E_{\mathrm{sx}}$ and $E_{\mathrm{sy}}$, of GaN nanosheets as a function of the (**a**) size of square NSs (current and Giannopoulos et al. [77] results) and (**b**) aspect ratio of NSs (current and Rouhi et al. [70] results).

Firstly, the current surface Young’s moduli, $E_{\mathrm{sx}}$ and $E_{\mathrm{sy}}$, of GaNNSs as a function of the nanosheet size (case of the square NSs, $L_{x}=L_{y}$) and aspect ratio, $L_{x}$/$L_{y}$, were compared with those available in the literature, as shown in Figure 12a,b. The analysis of these evolutions was carried out for nanosheets of comparable sizes and aspect ratios. As shown in Figure 12b, a set of GaNNSs with the same horizontal side length, $L_{x}$ ≈ 6 nm, was chosen; this value is similar to that used by Rouhi et al. [70], $L_{x}$ ≈ 5 nm.

In the present study, the evolution of the surface Young’s modulus for the zigzag configuration, $E_{\mathrm{sx}}$, with changing NS size was slightly higher for small nanosheets and then became stable with increasing NS size (Figure 12a), as in the case of Giannopoulos et al. [77], despite the differences in values. For the armchair configuration, the results pointed to a gradual increase in $E_{\mathrm{sy}}$ value when the NS size increased, contrary to Giannopoulos et al. [77]; also, in this case, the values were visibly different. With regard to the influence of the aspect ratio, Rouhi et al. [70] reported an increase in $E_{\mathrm{sx}}$ and $E_{\mathrm{sy}}$ moduli with increasing NS aspect ratio, $L_{x}$/$L_{y}$. These evolutions differ from the current ones, where the values of $E_{\mathrm{sx}}$ and $E_{\mathrm{sy}}$ demonstrated a nonsignificant increase and decrease, respectively, when $L_{x}$/$L_{y}$ increased (Figure 12b). Here too, the values were visibly different.

Table 8 summarizes the present results of the elastic moduli of square AlNNSs and GaNNSs and those available in the literature. Whenever possible, the NS size is specified in this table. The current Young’s and shear moduli calculated for AlN and GaN nanosheets of $L_{x}$ × $L_{y}$ ≈ 12 × 12 nm^2^, whose size is within the range of most NSs in Table 8, were chosen for comparison purposes. It is worth noting that solely theoretical (analytical and numerical) elastic moduli results are available in the literature.

**Figure 13:**
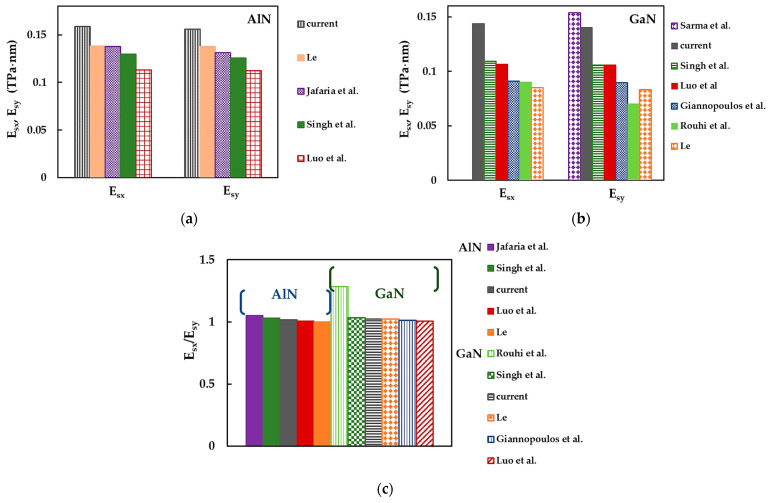
Surface Young’s moduli, $E_{\mathrm{sx}}$ and $E_{\mathrm{sy}}$, for (**a**) AlNNSs and (**b**) GaNNSs and (**c**) $E_{\mathrm{sx}}/E_{\mathrm{sy}}$ ratio for AlNNSs and GaNNSs obtained in the current study and reported by other authors [8,61,67,70,71,72,77] (see Table 8).

To facilitate a comparison of the current results and those available in the literature, presented in Table 8, the surface Young’s moduli, $E_{\mathrm{sx}}$ and $E_{\mathrm{sy}}$, and their ratio, $E_{\mathrm{sx}}/E_{\mathrm{sy}}$, are plotted in Figure 13.

As can be seen in Figure 13a and Table 8, the $E_{\mathrm{sx}}$ and $E_{\mathrm{sy}}$ moduli obtained for AlNNSs in the MD simulation study with Tersoff potential of Le [8] were in very good agreement with those evaluated by Jafaria et al. [61], who used ab initio calculations performed with the Quantum ESPRESSO (QE) package. The current $E_{\mathrm{sx}}$ and $E_{\mathrm{sy}}$ values were about 15% higher than those by Le [8] and Jafaria et al. [61], which in turn were nearly 5% higher than the $E_{\mathrm{sx}}$ and $E_{\mathrm{sy}}$ values calculated by Singh et al. [71], who used MS simulations employing TB potential function to describe the interactions between Al and N atoms. The lowest surface Young’s moduli in the zigzag and armchair directions were reported by Luo et al. [67], who used the Vienna ab initio simulation package (VASP) for ab initio DFT calculations. Although the studies of Jafaria et al. [61] and Luo et al. [67] implemented the Perdew–Burke–Ernzerhof (PBE) exchange–correlation functional and the projector-augmented wave (PAW) potentials for self-consistent total energy calculation and geometry optimization, the $E_{\mathrm{sx},y}$ values evaluated by Jafaria et al. [61] were nearly 20% higher than those by Luo et al. [67] due to the difference in the simulation package used.

With regard to GaNNSs, the current $E_{\mathrm{sx}}$ modulus remains the highest compared to those reported in the literature (see Figure 13b and Table 8). The $E_{\mathrm{sy}}$ value reported by Sarma et al. [72] was nearly 8% higher than that calculated in the present study. Singh et al. [71] and Luo et al. [67] obtained similar surface Young’s moduli values, contrary to what was observed for AlNNSs. $E_{\mathrm{sx}}$ and $E_{\mathrm{sy}}$ values by Luo et al. [67] and Singh et al. [71] were ≈ 20% higher than the respective moduli calculated by Giannopoulos et al. [77], who used spring elements to model the Ga–N bond under the NCM/MSM approach, and Rouhi et al. [70], who employed TB potential in their MD simulation study, with the exception of the $E_{\mathrm{sy}}$ value found in this last work. The lowest $E_{\mathrm{sx}}$ and $E_{\mathrm{sy}}$ values were reported by Le [8], despite his method providing satisfactory results for AlNNSs. The difference between the current surface Young’s moduli for the zigzag and armchair configurations and those evaluated by Giannopoulos et al. [77] can possibly be explained by the fact that they used a different elastic element, i.e., spring, to describe the Ga–N bond.

Most of the results shown in Figure 13c indicate that AlN and GaN nanosheets are transversely anisotropic, as shown by the ratio between the surface Young’s moduli, $E_{\mathrm{sx}}/E_{\mathrm{sy}}$. However, except for the findings by Rouhi et al. [70], who suggested strong anisotropy for GaNNSs, characterized by $E_{\mathrm{sx}}/E_{\mathrm{sy}}$ = 1.286, the values of $E_{\mathrm{sx}}/E_{\mathrm{sy}}$ presented in Figure 13c indicate a slight anisotropy of AlNNSs and GaNNSs. The values of $E_{\mathrm{sx}}/E_{\mathrm{sy}}$ were in the range of 1.019 (present study) to 1.050 (Jafaria et al. [61]) for AlNNSs and in the range of 1.013 (Giannopoulos et al. [77]) to 1.032 (Singh et al. [71]) for GaNNSs. Moreover, Le [8] reported $E_{\mathrm{sx}}/E_{\mathrm{sy}}$ ≈ 1 for AlNNSs, and Luo et al. [67] reported the same value for both AlNNSs and GaNNSs, which indicates isotropic behavior of the nanosheets. The current $E_{\mathrm{sx}}/E_{\mathrm{sy}}$ ratios for aluminum nitride and gallium nitride NSs were in a good concordance with those reported in the literature, indicating a mild NS anisotropy.

**Figure 14:**
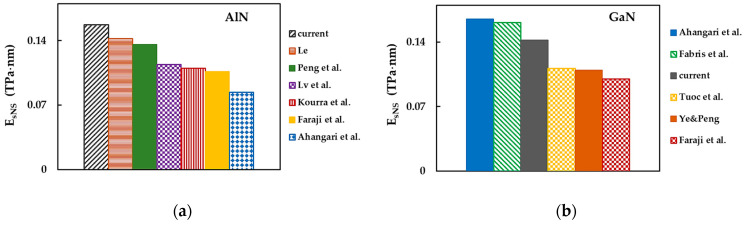
Comparison of the current surface Young’s modulus, $E_{\mathrm{sNS}}$, for (**a**) AlNNSs and (**b**) GaNNSs with those from [6,62,63,64,65,66,68,69].

Figure 14a,b compares the current average results of the surface Young’s modulus ($E_{\mathrm{sNS}}=\left(E_{\mathrm{sx}}+E_{\mathrm{sy}}\right)/2$) for AlNNSs and GaNNSs (considering the cases of NSs in Table 8), respectively, with those from the literature. For AlNNSs, the $E_{\mathrm{sNS}}$ value evaluated in the present work was ≈ 11% higher than that calculated by Le [76] using the analytical expression derived from the NCM/MSM approach (Figure 14a). In turn, $E_{\mathrm{sNS}}$ reported by Le [76] showed a good agreement (difference of about 4%) with the surface Young’s modulus obtained by Peng et al. [62], who performed their ab initio DFT calculations with the VASP code. These results were similar to $E_{\mathrm{sNS}}$ evaluated by Kourra et al. [63] and Lv et al. [64], who used the VASP and QE codes, respectively.

Faraji et al. [68] carried out their calculations with the VASP and obtained an $E_{\mathrm{sNS}}$ modulus of AlNNSs that was nearly 5% lower than those by Kourra et al. [63] and Lv et al. [64]. The lowest $E_{\mathrm{sNS}}$ value, as reported by Ahangari et al. [6], was evaluated using the Spanish Initiative for Electronic Simulations with Thousands of Atoms (SIESTA) code. In the works of Ahangari et al. [6], Peng et al. [62], Kourra et al. [63], Lv et al. [64], and Faraji et al. [68], the generalized gradient approximation (GGA) parameterized by the PBE functional was employed to describe the exchange–correlation energy, as implemented in the respective code. It is worth noting that a calculation approach similar to that in the aforementioned studies did not lead to comparable surface Young’s modulus results for AlNNSs (see Figure 14a).

As shown in Figure 14b for GaNNSs (in contrast to the case of AlNNSs), the highest surface Young’s modulus was reported by Ahangari et al. [6], whose $E_{\mathrm{sNS}}$ value was in very good agreement (difference of ≈ 2%) with that calculated by Fabris et al. [66]. These authors performed their ab initio DFT simulations using the CRYSTAL17 package combined with the Becke’s three-parameter Lee–Yang–Parr (B3LYP) functional to approximate the exchange–correlation energy. The $E_{\mathrm{sNS}}$ value reported by Fabris et al. [66] was about 14% higher than that evaluated in the present study. Tuoc et al. [65] and Ye and Peng [69] calculated similar values of $E_{\mathrm{sNS}}$, which were ≈ 29% lower than the current one. Tuoc et al. [65] used GCA-PBE parametrization within the VASP to this end, and Ye and Peng [69] employed ab initio DFT calculations via the five-order nonlinear elasticity (FONE) method. Finally, Faraji et al. [68] reported the $E_{\mathrm{sNS}}$ value to be ≈ 9% lower than those by Tuoc et al. [65] and Ye and Peng [69].

**Figure 15:**
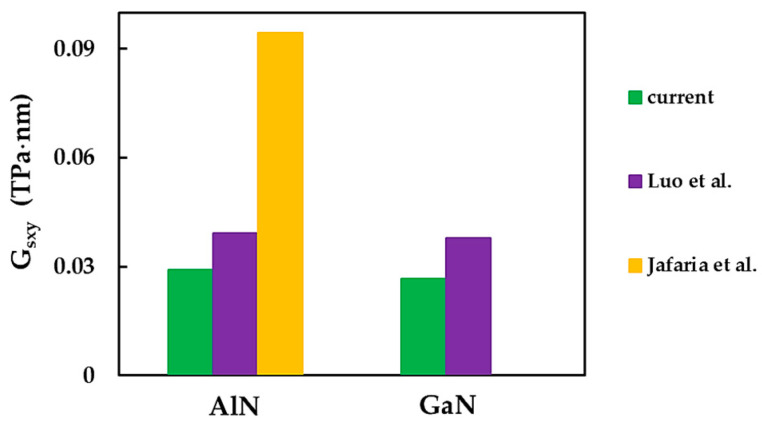
Comparison of the current surface shear modulus, $G_{\mathrm{sxy}}$, for AlNNSs and GaNNSs with those from [61,67].

As is evident from Table 8, available surface shear modulus results are scarce to date for AlN and GaN nanosheets. Figure 15 compares the current surface shear modulus, $G_{\mathrm{sxy}}$, for AlNNSs and GaNNSs with those reported in the literature. The discrepancy in the $G_{\mathrm{sxy}}$ values was obvious. The shear moduli of AlNNSs and GaNNSs in the current study were significantly lower than those obtained by Luo et al. [67] and Jafaria et al. [61]. A recent study [61] reported $G_{\mathrm{sxy}}$ for aluminum nitride NSs that exceeded the value reported in the present work by 200%.

It can be concluded from Table 8, Figure 13a,b, Figure 14 and Figure 15 that a considerable scattering of values of the surface Young’s and shear moduli was observed for AlNNSs and GaNNSs. Therefore, more theoretical results than those available so far are required to build a reliable benchmark for ascertaining the elastic moduli of aluminum nitride and gallium nitride NSs.

#### 3.3.2. Adjustment of Input Parameters for Numerical Simulation

**Figure 16:**
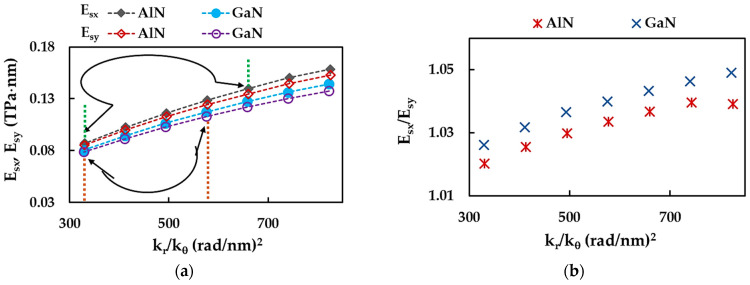
Evolutions of (**a**) the surface Young’s moduli, $E_{\mathrm{sx}}$ and $E_{\mathrm{sy}}$, and (**b**) their ratio, $E_{\mathrm{sx}}/E_{\mathrm{sy}}$, as a function of the ratio between the bond stretching and bond bending force constants, $k_{r}$/$k_{\theta }$, for AlNNSs and GaNNSs. The ranges of $k_{r}$/$k_{\theta }$ for which $E_{\mathrm{sx}}$ and $E_{\mathrm{sy}}$ are in better concordance with those from Table 8, are delimited by black arrows and green (AlN) and orange (GaN) dashed lines (**a**).

To obtain the surface Young’s moduli of AlNNSs and GaNNSs that show better agreement with those from the literature, a study was carried out on the influence of numerical simulation input parameters on the $E_{\mathrm{sx}}$ and $E_{\mathrm{sy}}$ Young’s moduli. The adjustment of the input parameters was performed by modifying the ratio between the bond stretching and bond bending force constants, $k_{r}$/$k_{\theta }$. In fact, the $k_{r}$/$k_{\theta }$ ratio was used in the calculation of the geometrical and elastic properties of the beam elements (see Table 5). For this purpose, the $k_{\theta }$ force constant remained the same and equal to that in Table 4. The $k_{r}$ force constant for each new simulation was calculated by $k_{r}^{\left(n\right)}={\;k}_{r}^{\left(n-1\right)}-0.1\cdot k_{r}^{\left(0\right)}$, where $k_{r}^{\left(0\right)}$ is the bond stretching constant from Table 4. AlNNSs and GaNNSs with the size 6.33 × 6.41 nm^2^ and 6.08 × 6.24 nm^2^, respectively, were chosen for numerical simulation. Figure 16a,b shows the surface Young’s moduli of AlNNSs and GaNNSs along the zigzag and armchair directions, $E_{\mathrm{sx}}$ and $E_{\mathrm{sy}}$ (Figure 16a), and their ratio, $E_{\mathrm{sx}}/E_{\mathrm{sy}}$ (Figure 16b), as a function of the $k_{r}$/$k_{\theta }$ ratio.

For AlNNSs and GaNNSs, the $E_{\mathrm{sx}}$ and $E_{\mathrm{sy}}$ moduli decreased with decreasing $k_{r}$/$k_{\theta }$ ratio, corresponding to the input parameters shown in Table 5 (see Figure 16a). A similar trend with a decrease in $k_{r}$/$k_{\theta }$ was observed for the evolutions of the $E_{\mathrm{sx}}/E_{\mathrm{sy}}$ ratio (Figure 16b). The ranges of the $k_{r}$/$k_{\theta }$ ratio, for which the $E_{\mathrm{sx}}$ and $E_{\mathrm{sy}}$ values were closer to those in Table 8 and Figure 13a and Figure 14, are shown in Figure 16a for AlNNSs and GaNNSs. To simplify understanding, the $k_{r}$ and $k_{\theta }$ force constants and the resulting numerical simulation input parameters, which led to the $E_{\mathrm{sx}}$ and $E_{\mathrm{sy}}$ values showing a good agreement with the literature results, are summarized in Table 9. The references reporting surface Young’s modulus that had good agreement with the current study are also presented in Table 9. The torsion resistance constant, $k_{\tau }$, was maintained the same as in Table 4. The diameter, d, Young’s modulus, $E_{b}$, and shear modulus, $G_{b}$, of the beam element were calculated by the expressions presented in Table 5. The Poisson’s ratio of the beam element, $\nu_{b}$, was assessed as follows [82]:

(17) $$\nu_{b}=\frac{k_{r}l^{2}-6k_{\theta }}{k_{r}l^{2}+18k_{\theta }},$$

where *l* is the beam length, which is equal to the Al(Ga)–N bond length.

As can be seen from Table 9, it is possible to decrease the surface Young’s modulus of AlNNSs and GaNNSs by decreasing the bond stretching force constant, $k_{r}$, which is required for calculating the input parameters of the numerical simulation. Although the proposed adjustment process brings the current $E_{\mathrm{sx}}$ and $E_{\mathrm{sy}}$ values closer to those reported in the literature, it also affects the outcomes regarding the NS anisotropy. The difference between the surface Young’s moduli for the zigzag and armchair configurations becomes more attenuated, and the $E_{\mathrm{sx}}/E_{\mathrm{sy}}$ ratio subsequently decreases with decreasing $k_{r}$ (see Figure 16b). Therefore, the effect on the nanosheet anisotropy should be taken into account when adapting the input parameters to change the resulting values of $E_{\mathrm{sx}}$ and $E_{\mathrm{sy}}$. Moreover, the use of a lower bond stretching force constant to calculate the numerical simulation input parameters results in thicker beam elements with lower elastic properties when compared with those of the initial model (see Table 9). This can affect the overall mechanical response of AlNNSs and GaNNSs and their subsequent analysis. Nevertheless, a detailed investigation of procedures for precisely tuning input parameters for numerical simulation is required and is planned for future work.

## 4. Conclusions

The surface elastic moduli of aluminum nitride and gallium nitride nanosheets with a large range of sizes and forms (from square to rectangular) were evaluated using numerical simulation based on the NCM/MSM approach. The present work is a systematic study and provides a robust finite element model of AlNNSs and GaNNSs having a wide range of aspect ratios, which allows an expeditious determination of their surface Young’s and shear moduli.

The evolutions of the current surface Young’s modulus, $E_{\mathrm{sx}}$ and $E_{\mathrm{sy}}$, and shear modulus, $G_{\mathrm{sxy}}$, of AlNNSs and GaNNSs with the nanosheet aspect ratio, $L_{x}$/$L_{y}$, was analyzed in terms of the NS forms. These evolutions ranged from NSs whose height was greater than their width ($L_{x}$/$L_{y}$ < 1) passing through square nanosheets ($L_{x}$/$L_{y}$ = 1) to NSs whose horizontal side was larger than the vertical ($L_{x}$/$L_{y}$ > 1).

The current results of the elastic moduli point to a slight anisotropy in AlNNSs and GaNNSs. The surface Young’s modulus in the zigzag direction, $E_{\mathrm{sx}}$, was greater than that in the armchair direction, $E_{\mathrm{sy}}$. For both AlNNSs and GaNNSs, the anisotropy ratio, $E_{\mathrm{sx}}/E_{\mathrm{sy}}$, was sensitive to the relationship between the NS side length sizes, $L_{x}$/$L_{y}$. For the square nanosheets, the value of $E_{\mathrm{sx}}/E_{\mathrm{sy}}$ was in good agreement with those reported in the literature.

Based on the current results, an analytical method was established and validated that allows accurate assessment of the surface Young’s and shear moduli of AlNNSs and GaNNSs without resorting to numerical simulation. To the best of our knowledge, such a methodology has not been previously proposed for aluminum nitride and gallium nitride nanosheets.

The results obtained substantially contribute to a benchmark for evaluating the elastic moduli of aluminum nitride and gallium nitride nanosheets by theoretical methods. This is an important outcome given that the development of this benchmark is still at an early stage.

An exploratory study on adjusting the input parameters for numerical simulation was performed, and its possible limitations were pointed out.

## Figures and Tables

**Table 1:** Values of the bond length of AlN and GaN nanostructures reported in the literature.

Compound	AlN	GaN
$a_{\mathrm{Al}(\mathrm{Ga})–N}$, nm	0.177 [5] 0.179 [3] 0.183 [6] 0.185 [7] 0.186 [8] 0.193 [9] 0.195 [10]	0.175 [6,11] 0.184 [5] 0.185 [3] 0.186 [7] 0.193 [8] 0.194 [9]

**Table 2:** Summarization of applications of 2D AlN and GaN nanostructures.

Compound	Applications
AlN	Deep ultraviolet light-emitting diode (DUV-LED) [12,13]; hydrogen storage [14]; toxic pollutants sensors [15]; cathode material for Al-ion batteries [16]
AlN, GaN	Toxic gas detectors [2,17,18,19,20,21]; field effect transistors (FETs) [1,22]; anode materials for Li- and Na-ion batteries [1,23,24,25,26] and Mg-ion batteries [27]; nanocarriers for drug delivery [28]
GaN	Light-emitting diodes (LEDs) [1,20,29,30]; piezoelectric devices [31,32]; photodetectors [33,34,35]

**Table 3:** Geometry of the studied single-layer AlNNSs and GaNNSs.

Compound	L_x_, nm	L_y_, nm	Aspect Ratio, $L_{x}{:L}_{y}$	Number of Elements	Number of Nodes
AlN	*3.17*	*3.11*	1.0 *	252	356
	6.33	3.11	2.0	492	706
	9.50	3.11	3.0	732	1056
	12.66	3.11	4.0	972	1406
	15.83	3.11	5.0	1212	1756
	3.17	6.41	0.5	504	722
	*6.33*	*6.41*	1.0 *	984	1432
	9.50	6.41	1.5	1464	2142
	12.66	6.41	2.0	1944	2852
	15.83	6.41	2.5	2424	3562
	3.17	9.70	0.3	756	1088
	6.33	9.70	0.7	1476	2158
	*9.50*	*9.70*	1.0 *	2196	3228
	12.66	9.70	1.3	2916	4298
	15.83	9.70	1.6	3636	5368
	3.17	12.99	0.2	1008	1454
	6.33	12.99	0.5	1968	2884
	9.50	12.99	0.7	2928	4314
	*12.66*	*12.99*	1.0 *	3888	5744
	15.83	12.99	1.2	4848	7174
	3.17	15.19	0.2	1176	1698
	6.33	15.19	0.4	2296	3368
	9.50	15.19	0.6	3416	5038
	12.66	15.19	0.8	4536	6708
	*15.83*	*15.19*	1.0 *	5656	8378
GaN	*3.38*	*3.32*	1.0 *	252	356
	6.08	3.32	1.8	444	636
	9.11	3.32	2.7	660	951
	12.14	3.32	3.7	876	1266
	15.17	3.32	4.6	1092	1581
	3.38	6.24	0.5	462	661
	*6.08*	*6.24*	1.0 *	814	1181
	9.11	6.24	1.5	1210	1766
	12.14	6.24	1.9	1606	2351
	15.17	6.24	2.4	2002	2936
	3.38	9.17	0.4	672	966
	6.08	9.17	0.7	1184	1726
	*9.11*	*9.17*	1.0 *	1760	2581
	12.14	9.17	1.3	2336	3436
	15.17	9.17	1.7	2912	4291
	3.38	12.09	0.3	882	1271
	6.08	12.09	0.5	1554	2271
	9.11	12.09	0.8	2310	3396
	*12.14*	*12.09*	1.0 *	3066	4521
	15.17	12.09	1.3	3822	5646
	3.38	15.02	0.2	1092	1576
	6.08	15.02	0.4	1924	2816
	9.11	15.02	0.6	2860	4211
	12.14	15.02	0.8	3796	5606
	*15.17*	*15.02*	1.0 *	4732	7001

* Square nanosheets (the aspect ratio $L_{x}{:L}_{y}$ ≈ 1.0) marked in underline and italics.

**Table 4:** Bond length, surface Young’s modulus, and Poisson’s ratio and the $k_{r}$, $k_{\theta }$, and $k_{\tau }$ force field constants for AlNNSs and GaNNSs.

Compound	$*\;a_{\mathrm{Al}(\mathrm{Ga})–N}$, nm	* E_s_, nN/nm	* ν	$k_{r}$, nN/nm	$k_{\theta }$, nN·nm/rad^2^	$k_{\tau }$, nN·nm/rad^2^
AlN	0.179	116	0.46	372	0.451	0.625
GaN	0.185	110	0.48	366	0.445	

* Values from Şahin et al. [3].

**Table 5:** Geometrical and elastic properties of the beams used as input parameters in FE simulations.

Compound	Diameter, d, nm	Formulation	Young’s Modulus, E_b_, GPa	Formulation	Shear Modulus, G_b_, GPa	Formulation	Poisson’s Ratio, $\nu_{b}$ [3]
AlN	0.1392	$d=4\sqrt{\frac{k_{\theta }}{k_{r}}}$	4374	$E_{b}=\frac{k_{r}^{2}l}{4\pi k_{\theta }}$	3032	$G_{b}=\frac{{k_{r}^{2}k}_{\tau }l}{8\pi k_{\theta }^{2}}$	0.46
GaN	0.1395		4437		3113		0.48

**Table 6:** Geometry of AlNNSs and GaNNSs chosen for validation purposes.

Compound	L_x_, nm	L_y_, nm	Aspect Ratio, $L_{x}{:L}_{y}$	Number of Elements	Number of Nodes
AlN	2.54	5.31	0.5	340	482
	5.07	5.31	1.0	660	954
	7.60	5.31	1.4	980	1426
	10.13	5.31	1.9	1300	1898
	15.83	5.31	3.0	2020	2960
GaN	2.70	8.58	0.3	510	727
	4.05	8.58	0.5	750	1083
	8.09	8.58	0.9	1470	2151
	12.14	8.58	1.4	2190	3219
	15.17	8.58	1.8	2912	4291

**Table 7:** Average differences between the $E_{\mathrm{sx}}$, $E_{\mathrm{sy}}$, and $G_{\mathrm{sxy}}$ values evaluated by the analytical expressions based on Equation (16) and those based on the data obtained from the FEA.

Compound	L_x_, nm	L_y_, nm	Average Difference, %		
			$E_{\mathrm{sx}}$, TPa·nm	$E_{\mathrm{sy}}$, TPa·nm	$G_{\mathrm{sxy}}$, TPa·nm
AlN	2.54	5.31	1.59	0.65	0.16
	5.07		0.96	1.00	0.27
	7.60		0.28	0.93	4.21
	10.13		0.28	0.07	2.17
	15.83		0.14	0.49	1.41
			* *0.65*	* *0.63*	* *1.65*
GaN	2.70	8.58	2.56	8.03	15.64
	4.05		3.37	5.57	1.91
	8.09		0.39	0.91	2.51
	12.14		3.29	2.06	0.76
	15.17		4.72	4.52	10.03
			* *2.86*	* *4.21*	* *6.17*

* Mean value of the average differences marked in italic.

**Table 8:** Comparison of the current Young’s and shear moduli results for AlNNSs and GaNNSs with those reported in the literature.

Reference	Method	Compound	$E_{\mathrm{sx}}$, TPa·nm	$E_{\mathrm{sy}}$, TPa·nm	$E_{x}$ $/E_{y}$	$G_{\mathrm{sxy}}$, TPa·nm	Size, nm^2^
Peng et al. [62]	ab initio DFT	AlN	0.136		–	–	–
Ahangari et al. [6]		AlN	0.084 ^1^		–	–	1.430 × 1.268
		GaN	0.165 ^1^				1.372 × 1.217
Jafaria et al. [61]		AlN	0.138	0.131	1.050	0.094	–
Luo et al. [67]		AlN	0.113	0.112	1.006	0.039	–
		GaN	0.107	0.106	1.007	0.038	
Tuoc et al. [65]		GaN	0.111 ^2^		–	–	–
Kuorra et al. [63]		AlN	0.110		–	–	
Lv et al. [64]		AlN	0.114		–	–	–
Fabris et al. [66]		GaN	0.161		–	–	–
Faraji et al. [68]		AlN	0.107 ^1^		–	–	–
		GaN	0.100 ^1^				
Ye and Peng [69]		AlN	0.136		–	–	–
		GaN	0.109				
Le [8]	MD: Tersoff potential	AlN	0.1381	0.1379	1.001		13.38 × 13.21
		GaN	0.085	0.083	1.022		13.85 × 13.68
Rouhi et al. [70]	MD: TB potential	GaN	0.090	0.070	1.286	–	5.371 × 5.132
Singh et al. [71]	MS: TB potential	AlN	0.130	0.126	1.030	–	9.948 × 11.69
		GaN	0.109	0.106	1.032		10.39 × 12.22
Sarma et al. [72]	MD: SW potential	GaN	–	0.154 ^3^	–	–	8.500 × 8.500
Le [76]	NCM/MSM: analytical solution	AlN	0.142		–	–	–
Giannopoulos et al. [77]	NCM/MSM: FE model, springs	GaN	0.0909 ^1^	0.0898 ^1^	1.013	–	10.00 × 10.00
Current study	NCM/MSM: FE model, beams	AlN	0.159	0.156	1.019	0.029	12.66 × 12.99
		GaN	0.144	0.140	1.024	0.027	12.14 × 12.09

^1^ Calculated from the Young’s modulus, $E_{\mathrm{NS}}$, using the equality $E_{\mathrm{sNS}}={\;E}_{\mathrm{NS}}{\cdot t}_{n}$ for AlNNS thicknesses of $t_{n}$ = 0.230 [6] and 0.213 [68] nm and for GaNNS thicknesses of $t_{n}$ = 0.226 [6], 0.229 [68], and 0.374 [77] nm. ^2^ Calculated from the second-order elastic constants, $C_{11}$ and $C_{12}$, by $E_{\mathrm{sNS}}=\left(C_{11}^{2}-C_{12}^{2}\right)/C_{11}$. ^3^ Calculated from the Young’s modulus, $E_{y}$, using the equality $E_{\mathrm{sy}}=E_{y}{\cdot t}_{n}$ for $t_{n}$ = 0.374 nm [72].

**Table 9:** Effect of input parameters on the surface Young’s modulus results of AlNNSs and GaNNSs and comparison with those reported by other authors.

Compound	Force Constants		Input Parameters				Results		Reference
	$k_{r}$, nN/nm	$k_{\theta }$, nN·nm/rad^2^	d	$E_{b}$, GPa	$G_{b}$, GPa	$\nu_{b}$	$E_{\mathrm{sx}}$, TPa·nm	$E_{\mathrm{sy}}$, TPa·nm	
AlN	298	0.451	0.1557	2799	1941	0.39	0.135	1.037	Le [8]; Jafaria et al. [61]; Peng et al. [62]
	260		0.1664	2143	1486	0.34	0.129	0.124	Singh et al. [71]
	223		0.1798	1575	1092	0.29	0.116	0.113	* Kourra et al. [63]; Lv et al. [64]; Luo et al. [67]; * Faraji et al. [68]
	186		0.1969	1094	758	0.23	0.103	0.100	* Kourra et al. [63]; * Faraji et al. [68]
	149		0.2201	700	485	0.16	0.087	0.085	Ahangari et al. [6]
GaN	256	0.445	0.1667	2174	1525	0.36	0.117	0.113	Tuoc et al. [65]
	220		0.1800	1597	1121	0.31	0.106	0.103	Luo et al. [67]; Faraji et al. [68]; Ye and Peng [69]; Singh et al. [71]
	183		0.1972	1109	778	0.25	0.094	0.091	Rouhi et al. [70]; Giannopoulos et al. [77]
	147		0.2205	710	498	0.18	0.081	0.079	Le [8]

* The surface Young’s modulus values are in satisfactory agreement with the current ones shown in both lines.

## Data Availability

The data presented in this study are available on request from the corresponding author after obtaining permission from an authorized person.

## Appendix A

**Table A1:** Surface Young’s modulus for the zigzag configuration, $E_{\mathrm{sx}}$, for AlNNSs and GaNNSs in Table 3.

AlN	$L_{y}$, nm					GaN	$L_{y}$, nm				
$L_{x}$ **, nm**	3.11	6.41	9.70	12.99	15.19	$L_{x}$ **, nm**	3.32	6.24	9.17	12.09	15.02
3.17	0.163	0.151	0.146	0.144	0.144	3.38	0.147	0.137	0.132	0.130	0.129
6.33	0.165	0.159	0.155	0.151	0.149	6.08	0.149	0.144	0.140	0.136	0.133
9.50	0.166	0.161	0.159	0.156	0.154	9.11	0.150	0.146	0.144	0.141	0.139
12.66	0.167	0.162	0.160	0.159	0.158	12.14	0.150	0.146	0.145	0.144	0.142
15.83	0.167	0.162	0.160	0.160	0.159	15.17	0.151	0.147	0.145	0.144	0.144

**Table A2:** Surface Young’s modulus for the armchair configuration, $E_{\mathrm{sy}}$, for AlNNSs and GaNNSs in Table 3.

AlN	$L_{y}$, nm					GaN	$L_{y}$, nm				
$L_{x}$ **, nm**	3.11	6.41	9.70	12.99	15.19	$L_{x}$ **, nm**	3.32	6.24	9.17	12.09	15.02
3.17	0.149	0.152	0.153	0.154	0.154	3.38	0.134	0.137	0.138	0.138	0.138
6.33	0.144	0.153	0.155	0.155	0.156	6.08	0.130	0.138	0.139	0.140	0.140
9.50	0.141	0.151	0.155	0.156	0.156	9.11	0.126	0.136	0.139	0.140	0.141
12.66	0.139	0.148	0.154	0.156	0.156	12.14	0.124	0.133	0.138	0.140	0.141
15.83	0.138	0.146	0.152	0.155	0.156	15.17	0.123	0.130	0.136	0.139	0.141

**Table A3:** Ratio between the surface Young’s moduli for zigzag and armchair configurations, $E_{\mathrm{sy}}$, for AlNNSs and GaNNSs in Table 3.

AlN	$L_{y}$, nm					GaN	$L_{y}$, nm				
$L_{x}$ **, nm**	3.11	6.41	9.70	12.99	15.19	$L_{x}$ **, nm**	3.32	6.24	9.17	12.09	15.02
3.17	1.090	0.994	0.956	0.940	0.934	3.38	1.095	1.005	0.961	0.941	0.930
6.33	1.146	1.039	1.002	0.973	0.959	6.08	1.147	1.049	1.008	0.976	0.955
9.50	1.183	1.067	1.026	1.003	0.991	9.11	1.188	1.074	1.032	1.007	0.986
12.66	1.198	1.091	1.042	1.019	1.008	12.14	1.209	1.101	1.049	1.024	1.007
15.83	1.205	1.111	1.058	1.030	1.019	15.17	1.219	1.123	1.067	1.036	1.019

**Table A4:** Surface shear modulus, $G_{\mathrm{sxy}}$, for AlNNSs and GaNNSs in Table 3.

AlN	$L_{y}$, nm					GaN	$L_{y}$, nm				
$L_{x}$ **, nm**	3.11	6.41	9.70	12.99	15.19	$L_{x}$ **, nm**	3.32	6.24	9.17	12.09	15.02
3.17	0.029	0.011	0.006	0.003	0.002	3.38	0.026	0.012	0.006	0.004	0.003
6.33	0.046	0.029	0.018	0.012	0.009	6.08	0.039	0.025	0.016	0.011	0.008
9.50	0.052	0.040	0.029	0.021	0.017	9.11	0.044	0.035	0.026	0.019	0.015
12.66	0.056	0.046	0.037	0.029	0.025	12.14	0.047	0.040	0.033	0.027	0.021
15.83	0.059	0.049	0.042	0.035	0.031	15.17	0.050	0.043	0.038	0.032	0.027

## Appendix B

**Table A5:** Fitting parameters of Equation (16) to determine the surface elastic moduli of AlN nanosheets.

Parameter	$E_{\mathrm{sx}}$, TPa·nm	$E_{\mathrm{sy}}$, TPa·nm	$G_{\mathrm{sxy}}$, TPa·nm
$P_{00}$	0.171226860171277	0.144651640333808	0.026970084065154
$P_{10}$	−0.006360347217088	0.002967422265006	−0.007902381722436
$P_{01}$	0.002279765484141	−0.001843434579208	0.009085303791427
$P_{20}$	0.000363968852232	−0.000293419339182	0.000426608771576
$P_{11}$	0.000229759364727	0.000292133965245	−0.000092489299104
$P_{02}$	−0.000209500336416	0.000012728748904	−0.000545715913699
$P_{30}$	−0.00000746339864	0.000007833660811	−0.000003678537337
$P_{21}$	−0.000007518745212	−0.000005131252802	−0.000017320436461
$P_{12}$	−0.000000604912003	−0.000005767443066	0.000022679820597
$P_{03}$	0.000004471395921	0.000000523690948	0.00000856130266
R^2^	0.9900	0.9818	0.9968

**Table A6:** Fitting parameters of Equation (16) to determine the surface elastic moduli of GaN nanosheets.

Parameter	$E_{\mathrm{sx}}$, TPa·nm	$E_{\mathrm{sy}}$, TPa·nm	$G_{\mathrm{sxy}}$, TPa·nm
$P_{00}$	0.155842264053617	0.126851645283434	0.031381726719575
$P_{10}$	−0.006331424238629	0.003752888683127	−0.008841303632553
$P_{01}$	0.002195242203642	−0.001460259028607	0.006296030975982
$P_{20}$	0.000368839364287	−0.000407373789112	0.000575024580332
$P_{11}$	0.000259013009783	0.000322511258052	0.000006526629769
$P_{02}$	−0.000214522770561	−0.000048349919454	−0.000285511583613
$P_{30}$	−0.000008401279696	0.000012092804498	−0.000009642448834
$P_{21}$	−0.000007343339536	−0.000005998213089	−0.00001933757749
$P_{12}$	−0.000001894734595	−0.000006015715182	0.000019453550129
$P_{03}$	0.0000047883614	0.000002658775622	−0.000000144991164
R^2^	0.9873	0.9822	0.9969
